# Differential gene expression in neonatal calf muscle tissues from Hanwoo cows overfed during mid to late pregnancy period

**DOI:** 10.1038/s41598-024-74976-3

**Published:** 2024-10-07

**Authors:** Borhan Shokrollahi, Myungsun Park, Youl-Chang Baek, Shil Jin, Gi-Suk Jang, Sung-Jin Moon, Kyung-Hwan Um, Sun-Sik Jang, Hyun-Jeong Lee

**Affiliations:** 1 Hanwoo Research Institute, National Institute of Animal Science, Pyeongchang, 25340 Korea; 2 Animal Nutrition and Physiology Division, National Institute of Animal Science, Rural Development Administration, 55365 Wanju, Korea

**Keywords:** Maternal nutrition, Skeletal muscle development, Metabolic programming, Transcriptomic analysis, Beef cattle metabolism, Biological techniques, Cancer, Developmental biology, Genetics

## Abstract

Maternal nutrition significantly influences fetal development and postnatal outcomes. This study investigates the impact of maternal overfeeding during mid to late pregnancy on gene expression in the round and sirloin muscles of Hanwoo neonatal calves. Eight cows were assigned to either a control group receiving standard nutrition (100%) or a treated group receiving overnutrition (150%). After birth, tissue samples from the round and sirloin muscles of neonatal calves were collected and subjected to RNA sequencing to assess differentially expressed genes (DEGs). RNA sequencing identified 43 DEGs in round muscle and 15 in sirloin muscle, involving genes related to myogenesis, adipogenesis, and energy regulation. Key genes, including PPARGC1A, THBS1, CD44, JUND, CNN1, ENAH, and RUNX1, were predominantly downregulated. Gene ontology (GO) enrichment analyses revealed terms associated with muscle development, such as “biological regulation,” “cellular process,” and “response to stimulus.” Protein-protein interaction networks highlighted complex interactions among DEGs. Random Forest analysis identified ARC, SLC1A5, and GNPTAB as influential genes for distinguishing between control and treated groups. Overall, maternal overnutrition during mid-to-late pregnancy results in the downregulation of genes involved in muscle development and energy metabolism in neonatal Hanwoo calves. These findings provide insights into the molecular effects of maternal nutrition on muscle development.

**Supplementary Information:**

The online version contains supplementary material available at 10.1038/s41598-024-74976-3.

## Introduction

Investigating the complex mechanisms of mammalian muscle development at the molecular level is pivotal for enhancing muscle growth and applying genetic knowledge to improve beef production. Recent advancements in sequencing technologies, such as RNA-Seq (RNA sequencing), have significantly enhanced our ability to study gene expression, exploring the fundamental molecular mechanisms behind the growth, metabolism, and disease resistance in livestock^1^.

Fetal tissue development in cattle, intricately influenced by maternal nutrition, plays a crucial role in determining growth performance and meat quality. Emerging evidence suggests that strategic nutritional interventions during pregnancy can significantly impact fetal development, emphasizing the importance of adequate nutrition and avoiding excess during mid-to-late pregnancy, which greatly affects postnatal outcomes^2,3^.

As cows approach late pregnancy, their nutritional requirements increase, highlighting the critical nature of this period for fetal muscle development and setting the stage for effective ‘fetal programming’. This practice has the potential to enhance muscle fiber number and mass, and alter energy metabolism in skeletal muscles—a key determinant for cattle, where muscle fibers are established prenatally and remain constant postnatally^4,5^. Furthermore, the nutritional adequacy during this period affects prenatal physiological processes, potentially influencing postnatal growth and meat production outcomes^4,6,7^.

Transcriptome studies in cattle during mid-to-late pregnancy have provided insights into how maternal nutrition influences offspring muscle development, specifically highlighting genes and pathways involved in muscle differentiation, growth regulation, and immune response modulation^8,9^. These findings underscore the pivotal role of maternal diet in shaping muscle architecture and function in neonatal calves^10^.

Nutrition management of dams during mid-to-late pregnancy is crucial for modulating gene expression associated with myogenesis, adipogenesis, and energy homeostasis^11^. Nutritional balance during this period directly influences developmental pathways controlling muscle formation and fat deposition in offspring, with both undernutrition and overnutrition having distinct effects^11,12^. Optimal maternal nutrition not only increases muscle fibers and mass but also intramuscular adipocytes, contributing to improved beef quality^13,14^. Carvalho, et al^15^. and Duarte, et al^16^. have shown that providing supplemental protein to dams during mid-pregnancy impacts energy metabolism and supports skeletal muscle growth. Ahn, et al^17^. further demonstrated that increased protein intake during pregnancy enhances fetal growth, leading to improved birth weight and carcass traits in Hanwoo male calves. Additionally, our earlier report demonstrated that maternal overnutrition enhances specific anatomical and morphometric traits in Hanwoo neonatal calves, with no observed changes in carcass traits^18^. Furthermore, our recent findings have shown that maternal overnutrition during mid-to-late gestation significantly affects the transcriptomic responses of subcutaneous adipose tissue and liver in newborn Hanwoo calves^19^.

In this study, we hypothesized that overnutrition of Hanwoo mothers during mid-to-late pregnancy could alter differences in gene expression within pathways associated with muscle development and marbling in the muscles of newborn calves. Our goal was to deepen the understanding of the individual genes and pathways influenced in the round and sirloin muscles of neonatal calves born to mothers receiving supplementary nutrition during mid-to-late pregnancy.

## Materials and methods

### Animals, diets and management

This study was conducted at the Hanwoo Research Institute, part of the National Institute of Animal Science, over a 10-month period from June 2021 to April 2022. A total of eight pregnant Hanwoo cows were randomly selected for the study. Before artificial insemination, all cows were managed uniformly and fed a standardized diet (Table 1) according to the Korean Feeding Standard for Hanwoo^20^. After insemination, the cows were divided into two dietary groups, each consisting of four cows. At the start (from artificial insemination to 3 months post-insemination) and middle of pregnancy (approximately days 90 to 180 of pregnancy), there were no significant differences in age, weight, and body condition score (BCS) between the groups. However, the overfed cows showed an increase in BCS during late pregnancy^18^. Each cow required an average of 1.5 inseminations per pregnancy, which was confirmed 90 days post-insemination using a pregnancy diagnosis kit and manual verification.

**Table 1:** Nutritional composition of feeds for Hanwoo cattle in the study.

Item	Concentrate	Rice straw
Dry matter (%)	88.43	97.87
	**% of dry matter**	
Crude Protein	16.43	6.22
TDN^1^	75.40	60.64
NDF^2^	35.64	71.47
ADF^3^	13.83	38.56
Ash	7.55	11.31
Ether extract	3.42	0.64
Crude fiber	8.14	29.99
Lignin	2.88	4.50

^1^Total digestible nutrient^2^;Neutral detergent fiber^3^;Acid detergent fiber.

Initially, both groups received the same diet of rice straw and concentrate. The control group was given 3 kg of concentrate and 5 kg of rice straw daily throughout pregnancy. This diet meets the Korean Feeding Standards for Hanwoo, specifying a Dry Matter Intake (DMI) of 4.71 kg, Total Digestible Nutrients (TDN) of 2.12 kg, and Crude Protein (CP) of 0.47 kg for a 400 kg cow. In our experiment, the control group’s intake was set at a DMI of 6.07 kg, TDN of 4.07 kg, and CP of 0.65 kg, reflecting typical feeding levels for pregnant cows according to these standards^20^.

The treatment group, designed to simulate overnutrition, received 4.5 kg of concentrate and 6.5 kg of rice straw daily during mid-to-late gestation (from day 90 to the end of pregnancy), representing a 150% increase in feed. This group’s intake was set at a DMI of 8.42 kg, TDN of 5.69 kg, and CP of 0.93 kg, resulting in a net energy for maintenance (NEm) of 9.56 Mcal, compared to 6.68 Mcal for the control group. This strategy aimed to study the effects of overfeeding on fetal development, focusing on metabolic and physiological changes due to increased nutritional intake. The dietary compositions were analyzed following AOAC guidelines^21^.

### Neonatal calves slaughtering and muscles tissue sampling and preservation

Following birth, neonatal male calves (average weight 30.9 kg) were immediately separated from their mothers to ensure they did not ingest any colostrum, as required by the study’s experimental design. The calves were then humanely euthanized using a protocol approved by the Institutional Animal Care and Use Committee (IACUC) under protocol number NIAS 2020-095. This involved administering pentobarbital intravenously at a dose of 100 mg/kg body weight^22^ to ensure rapid and humane cessation of life functions. This method, followed by exsanguination, was carefully chosen to meet international standards for ethical animal treatment reducing distress to the animals and preserving tissue integrity essential for our molecular analyses. Post-mortem examination, specific muscle specimens (about 50–100 g) from both the round and sirloin regions were meticulously excised. These samples were immediately stored in liquid nitrogen before being transported to a controlled laboratory environment. There, they were stored at a sub-zero temperature of -80 °C to halt any biochemical activity and maintain tissue integrity until detailed molecular analysis. This preservation strategy was employed to ensure the samples remained viable for subsequent analyses.

### RNA extraction, quality control, and sequencing

Total RNA was isolated from neonatal calf muscle samples using the TRIzol reagent to minimize degradation and contamination. Following extraction, RNA samples were immediately stored at -80 °C to maintain their integrity until further processing. The integrity and quality of RNA were stringently assessed using a TapeStation RNA screentape (Agilent, #5067–5576). From this quality and quantity evaluation, only high-quality RNA preparations, with RNA Integrity Number (RIN) greater than 7.0, were used for RNA library construction. Specifically, the RIN for these samples ranged from 7.0 to 8.0, indicating good RNA integrity suitable for RNA sequencing. Subsequently, a library was independently prepared with 0.5 µg of total RNA for each sample by Illumina TruSeq Stranded Total RNA Library Prep Globin Kit (Illumina, Inc., San Diego, CA, USA, #20020613). The first step in the workflow involved removing the rRNA in the total RNA using the Ribo-Zero rRNA Removal Kit (Illumina, Inc., San Diego, CA, USA). Following this step, the remaining RNA is fragmented into small pieces using divalent cations under elevated temperature. The cleaved RNA fragments were then copied into first-strand cDNA using SuperScript II reverse transcriptase (Invitrogen, #18064014) and random primers. This was followed by second-strand cDNA synthesis using DNA Polymerase I, RNase H, and dUTP. These cDNA fragments then go through an end repair process, the addition of a single ‘A’ base, and then ligation of the adapters. The products were then purified and enriched with PCR to create the final cDNA library. The libraries were quantified using KAPA Library Quantification kits for Illumina Sequencing platforms according to the quantitative PCR (qPCR) Quantification Protocol Guide (KAPA BIOSYSTEMS, #KK4854) and qualified using the TapeStation D1000 ScreenTape (Agilent Technologies, #5067–5582). Indexed libraries were then submitted to an Illumina NovaSeq (Illumina, Inc., San Diego, CA, USA), and the paired-end (2 × 100 bp) sequencing was performed by the TNT Research Incorporated.

### Post-sequencing processing and transcriptomic analysis

The raw sequencing reads were processed to remove low-quality sequences and adapters, and the quality metrics, including base quality, sequence content, and read length, were assessed using FastQC v0.11.7. Before performing RNA sequence mapping, adapter sequence was removed using the Trimmomatic v0.39 program, and reads were trimmed by removing bases with a base quality less than 3, a sliding window trim size of 4, a mean quality of 15, and a minimum length of 36 bp. To ensure the removal of any residual rRNA reads, SortMeRNA was used post-sequencing to filter out rRNA sequences from the data. After preprocessing, the cleaned reads were indexed and aligned to the reference bovine genome using HISAT2 version 2.1.0 and Bowtie2 version 2.3.4.1. Following alignment, transcript assembly and abundance estimation were conducted with StringTie version 2.1.3b. StringTie provided quantification metrics such as Fragments Per Kilobase of transcript per Million mapped reads (FPKM) and Transcripts Per Million (TPM), which are essential for subsequent differential expression analysis. The expression profiles obtained from StringTie were then used for additional analyses to identify DEGs. Statistical hypothesis testing was performed to filter DEGs between the control and treated groups.

### Differential expression and functional enrichment

Statistical analyses of differential gene expression were performed using DESeq2 v1.24.0 with raw counts as input, identifying statistically significant changes in expression at an adjusted p-value < 0.05. Visualization techniques, including volcano plots and heatmaps generated through R packages like ggplot2 and pheatmap, facilitated the exploration of gene expression patterns and clustering across samples. Further, functional enrichment analysis using g: Profiler shed light on the biological implications of the DEGs, pinpointing their involvement in specific biological processes, cellular components, molecular functions, and KEGG pathways.

Principal Component Analysis (PCA) was conducted to identify patterns in the gene expression data and to visualize the variation between the control and treated groups. PCA plots were generated using the factoextra package in R, which provided a clear graphical representation of the separation between the groups. Pearson correlation analysis was performed to assess the relationships between samples. The correlation matrices were visualized to display the strength of the relationships, with higher correlations indicated by darker shades. These analyses helped to confirm the distinct gene expression patterns between the control and treated groups.

### Validation of gene expression data

To validate the RNA sequencing outcomes, we employed qPCR to assess the expression levels of seven DEGs. Therefore, total RNA was reverse transcribed into cDNA utilizing the PrimeScript™ RT Reagent Kit with gDNA Eraser, following the protocol provided by the manufacturer (TaKaRa, Dalian, China). The qPCR analysis was executed using the SYBR^®^ Green Realtime PCR Master Mix (code No. QPK-201, 201 × 5; Toyobo, Osaka, Japan) on a Bio-Rad CFX96 Real-Time PCR System (Hercules, CA, United States), with primer details listed in Table 2. The amplification conditions included an initial denaturation at 95 °C for 30 s, followed by 39 cycles of denaturation at 95 °C for 5 s and annealing at 55 °C for 40 s. Additionally, GAPDH was co-amplified to normalize the expression levels of the target genes. The relative expression levels of the DEGs were quantified using the 2^(-ΔΔCt) method^23^.

**Table 2:** List of selected primers for qPCR validation of RNA sequencing results of round and sirloin samples.

Gene	Primer sequence	Product length	Accession number
LOC104970902	F: GCTCACAGGACTAGCAGGAA R: AGCAATGGCTTTGTGGAGTG	107	XR_001500607
PPARGC1A	F: TGCTCTGTGTCACTGTGGATT R: AACCAGAGCAGCACACTCG	123	NM_177945.3
FOSL1	F: TGTTCCGAGACTACGGGGA R: AGGTGGAACTTCTGCTGGC	100	NM_001205985.1
THBS1	F: AGTGTCGCTGCCAGAACTC R: CAGCCATCGTCTGCAGAGTC	133	NM_174196.1
CNN1	F: GAGGTCAAGAACAAGCTGGC R: CGTCCATGAAGTTGTTGCCG	109	NM_001046379.1
SGMS2	F: GTGGGGCGCAGATTCTTTTT R: GAGTCTCCGTTGAGCTTTGGA	125	NM_001205877.3
GAPDH*	F: TGACCCCTTCATTGACCTTC R: GATCTCGCTCCTGGAAGATG	143	NM_001190390.1

### Protein-protein Interaction (PPI) analysis

To elucidate the complex interactions between proteins encoded by the DEGs (adjusted p-value < 0.05) identified in our study, we conducted a PPI analysis using the STRING database (version 12.0). STRING is a comprehensive resource for exploring predicted and known interactions among proteins, integrating data from various sources including experimental data, computational prediction methods, and public text collections. For the PPI analysis, the list of DEGs from both round (40) and sirloin (7) muscle tissues was input into STRING. We used the default settings to explore interactions, including direct (physical) and indirect (functional) associations. The database provides a confidence score for each interaction, which is based on the evidence supporting that interaction. We set a minimum required interaction score of 0.4 (medium confidence) to ensure the relevance and reliability of the interactions displayed.

### Identification of influential genes using random forest

To identify the most influential genes on muscle tissue characteristics, we employed the Random Forest algorithm using the random forest package in R (version 4.0.3). The Random Forest algorithm was chosen for its robustness and accuracy in handling complex biological data. It effectively manages large datasets with many variables and provides reliable feature importance metrics, making it ideal for identifying key genes in our study. This machine learning method constructs multiple decision trees and outputs the mode (classification) or mean prediction (regression) of the trees. Our dataset comprised 44 DEGs with an adjusted p-value < 0.05. We used supervised training with the experimental group (treated or control) as the predicted variable, splitting the data into 70% for training and 30% for validation. A 10-fold cross-validation was employed to ensure robustness and mitigate overfitting, repeating this process ten times. The analysis used 500 trees to ensure stability, with the number of variables at each split set to the square root of the number of DEGs. We measured the Mean Decrease in Accuracy (MDA) for each gene to quantify its importance in classification. Model performance was evaluated by calculating the proportions of correctly and incorrectly classified instances and overall accuracy, supplemented by a confusion matrix detailing true positive, false positive, true negative, and false negative classifications. These results provided a comprehensive assessment of the model’s predictive performance and its reliability in identifying influential genes.

### Statistical analysis

The study analyzed differential gene expression in neonatal Hanwoo calf muscles resulting from maternal overnutrition. The RNA sequencing data were processed using FastQC for quality control, Trimmomatic for trimming adapters and low-quality sequences, and HISAT2 and Bowtie2 for alignment to the reference genome. Differential gene expression analysis was performed using DESeq2, identifying 43 DEGs in round muscle and 15 in sirloin muscle, with significance set at an adjusted p-value < 0.05. GO and KEGG pathway enrichment analyses were conducted using g: Profiler, with DEGs at an adjusted p-value < 0.2 to generate more terms. Protein-protein interactions were analyzed using the STRING database, and key gene influences were examined using the Random Forest analysis by the randomForest package with the significant DEGs (adjusted p-value < 0.05). qPCR was used to validate the RNA-Seq data. For comparisons of means between the two groups, the t-test was applied using R, and fold changes are reported as Means ± SEM.

## Results

### RNA-sequencing analysis

Sixteen muscle samples from neonatal Hanwoo calves (both round and sirloin) were analyzed to study the effects of maternal overnutrition during mid to late pregnancy. The RIN for these samples ranged between 7.0 and 8.0, showing good RNA quality. RNA sequencing yielded a total of 888.2 million paired-end reads. Each round muscle sample produced approximately 54.5 million reads, and each sirloin muscle sample produced about 56.5 million reads. For both muscle types, 99.1% of the reads matched the bovine genome.

### Differential gene expression analysis

In this study, differential gene expression analysis was conducted on 34,109 genes across both round and sirloin muscle samples of neonatal calves. A significant proportion of genes, specifically 16,897 from round muscle and 16,885 from sirloin tissue, displaying FPKM values below the threshold of 0.1, were excluded from the dataset^24,25^. The comprehensive tabulation of all genes is presented in Table S1.

**Fig. 1:**
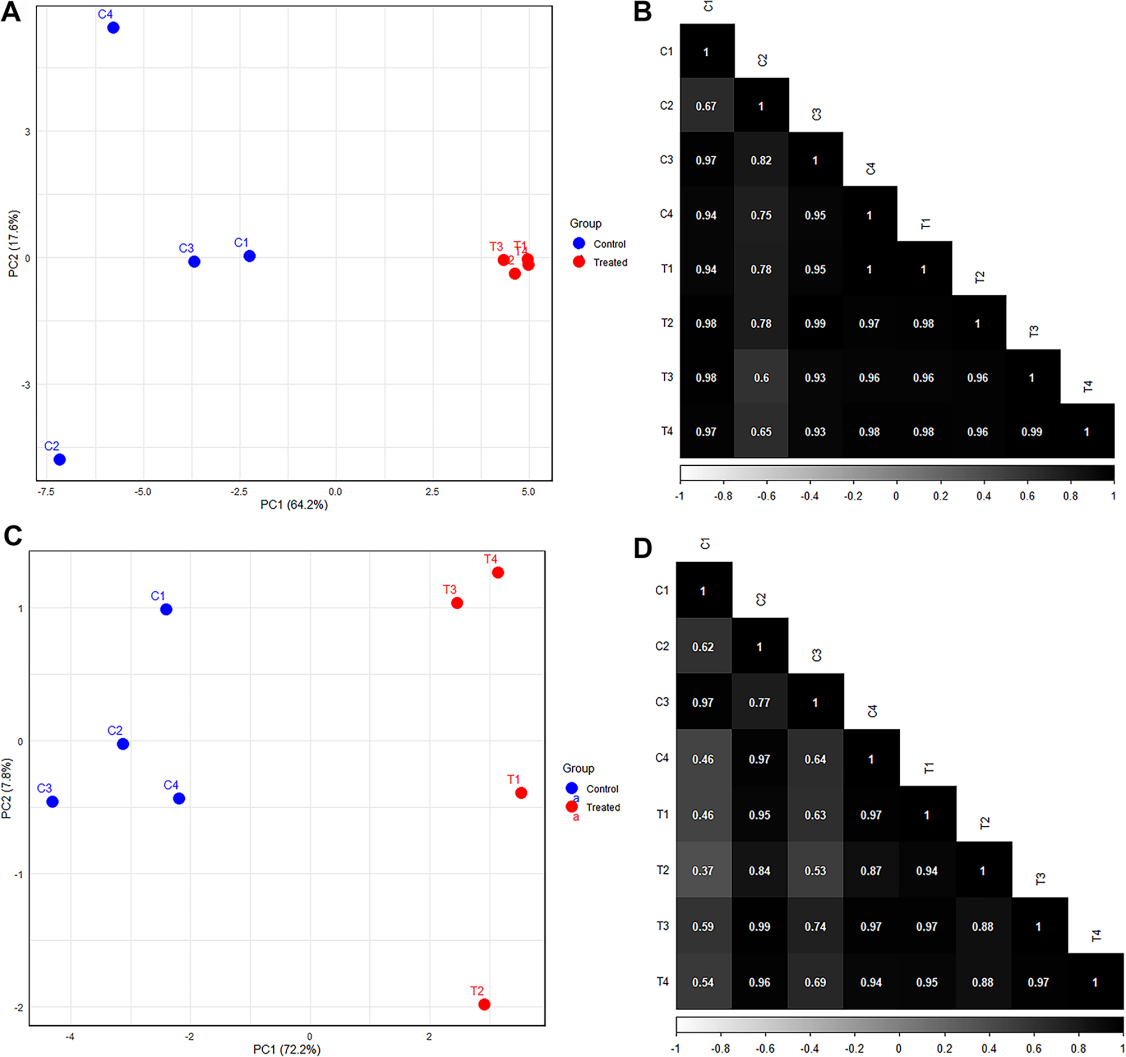
Principal Component Analysis (PCA) and Pearson correlation analysis of gene expression profiles in round and sirloin muscle tissues of neonatal Hanwoo calves. (**A**) PCA plot for round muscle tissue samples. Each point represents an individual sample, with blue points indicating control samples (C1, C2, C3, C4) and red points indicating treated samples (T1, T2, T3, T4). The PCA plot shows clear separation between control and treated groups. (**B**) The matrix shows the correlation coefficients between pairs of samples, with higher correlations indicated by darker shades. Control and treated samples exhibit high intra-group correlations, while inter-group correlations are lower. (**C**) PCA plot for sirloin muscle tissue samples. Each point represents an individual sample, with blue points indicating control samples and red points indicating treated samples. The PCA plot shows clear separation between control and treated groups. (**D**) Pearson correlation matrix for sirloin muscle tissue samples. The matrix shows the correlation coefficients between pairs of samples, with higher correlations indicated by darker shades. Control and treated samples exhibit high intra-group correlations, while inter-group correlations are lower.

Additionally, we performed PCA and Pearson correlation analysis to further validate our findings. The PCA plots for the round (Fig. 1A) and sirloin muscle samples (Fig. 1C) clearly distinguish between the control and treated groups, indicating significant variance in gene expression profiles due to maternal overnutrition. However, some variability was observed within the control group samples. For the round muscle (Fig. 1A), while the treated group samples (T1, T2, T3, T4) form a distinct cluster, one control sample (C2) appears to be an outlier, clustering separately from the other control samples (C1, C3, C4). This suggests that while there is a general distinction between the treated and control groups, there may be variability within the control samples that may affect the clustering. In the sirloin muscle samples (Fig. 1C), the PCA plot shows a more distinct separation between the treated and control groups. The treated samples (T1, T2, T3, T4) cluster tightly together, whereas the control samples (C1, C2, C3, C4) form a separate group but with a broader spread, indicating some variability within the group. The Pearson correlation analysis for the round muscle samples (Fig. 1B) shows high correlations between most samples, except for sample C2, which has lower correlations with the other control samples, further supporting its status as an outlier. The correlation matrix for the sirloin muscle samples (Fig. 1D) indicates high intra-group correlations among treated samples and some variability among control samples. These observations suggest that while there is a general distinction between treated and control groups in gene expression profiles, variability within control groups must be considered.

Further analysis on the round muscle tissue revealed significant differential expression in 855 genes, with a p-value < 0.05. Among these, 321 genes were found to be upregulated, while 534 genes showed downregulation. For sirloin, a total of 677 genes met the established criteria (p-value < 0.05), with 336 genes upregulated and 341 downregulated.

### Visualization of DEGs

**Fig. 2:**
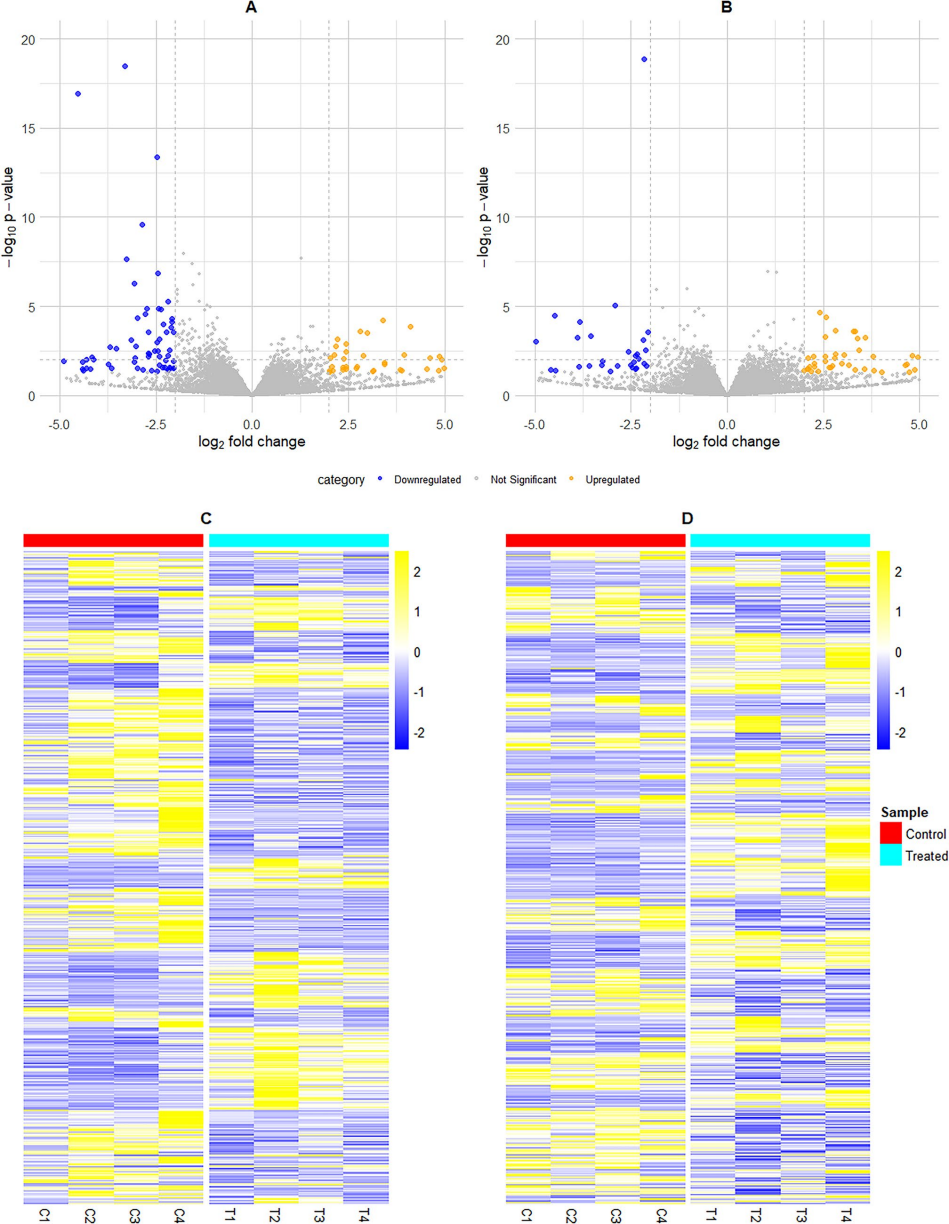
Visualization of differentially expressed genes (DEGs) due to maternal overfeeding during mid to late pregnancy in the muscle tissues of neonatal Hanwoo calves. (**A**) Volcano plot for round muscle tissue. The Y-axis represents the -log10 p-values, while the X-axis shows the log2 fold change (FC). Blue points on the left indicate significantly downregulated DEGs, and orange points on the right indicate significantly upregulated DEGs (p-value < 0.05). Grey points represent non-significant DEGs. (**B**) Volcano plot for sirloin muscle tissue. Similar to panel A, the Y-axis represents the -log10 p-values, and the X-axis shows the log2 fold change (FC). Blue points indicate significantly downregulated DEGs, and orange points indicate significantly upregulated DEGs (p-value < 0.05). Grey points represent non-significant DEGs. (**C**) Heatmap depicting DEGs for round muscle tissue. Columns represent individual samples: control samples (C1, C2, C3, C4) and treated samples (T1, T2, T3, T4). Colors range from blue (downregulated) to yellow (upregulated) based on the Z-score of normalized expression values. (**D**) Heatmap depicting DEGs for sirloin muscle tissue. Columns represent individual samples: control samples and treated samples. Colors range from blue (downregulated) to yellow (upregulated) based on the Z-score of normalized expression values.

The volcano plots showing the effect of maternal overfeeding on gene expression in the round and sirloin muscles of Hanwoo neonatal calves are illustrated in Fig. 2A and B, respectively. These plots illustrate the distribution of DEGs, highlighting significant upregulated and downregulated genes. The distinct gene expression profiles of these DEGs are further visualized in the heatmaps in Fig. 2C and D. Figure 2C shows the heatmap for the round muscle, while Fig. 2D displays the heatmap for the sirloin muscle.

Notably, 43 DEGs in the round muscle and 15 DEGs in the sirloin muscle exhibited significant differential expression (adjusted p-value < 0.05) between the two study groups. In the round muscle, there were 4 upregulated and 39 downregulated DEGs (Table 3). In contrast, in the sirloin muscle, 6 DEGs were upregulated, and 9 were downregulated (Table 4).

**Table 3:** List of round muscle DEGs with their statistical and fold change information.

Gene_Symbol	Description	logFCe	Adj_P_Value	Expression_Status
TNMD	Tenomodulin	−7.381	6.49e-03	Downregulated
LOC112446708	lncRNA	−7.372	1.56e-02	Downregulated
LOC784052	40 S ribosomal protein S26-like	−6.626	9.04e-08	Downregulated
ARC	Activity regulated cytoskeleton associated protein	−5.421	1.34e-09	Downregulated
LOC101904275	Pseudogene	−5.361	3.90e-04	Downregulated
FOSL1	FOS like 1, AP-1 transcription factor subunit	−4.530	9.79e-14	Downregulated
THBS1	Thrombospondin 1	−3.309	5.46e-15	Downregulated
RUNX1	Runt related transcription factor 1	−3.270	3.82e-05	Downregulated
GIPC2	GIPC PDZ domain containing family member 2	−3.053	5.70e-04	Downregulated
BRD3OS	BRD3 opposite strand, transcript variant X1	−2.976	2.25e-02	Downregulated
SGMS2	Sphingomyelin synthase 2	−2.857	6.37e-07	Downregulated
IPMK	Inositol polyphosphate multikinase	−2.786	1.49e-02	Downregulated
PPARGC1A	PPARG coactivator 1 alpha	−2.733	8.56e-03	Downregulated
ENAH	ENAH, actin regulator	−2.468	1.72e-10	Downregulated
ARL4C	ADP ribosylation factor like GTPase 4 C	−2.445	1.90e-04	Downregulated
DLK2	Delta like non-canonical Notch ligand 2	−2.422	8.56e-03	Downregulated
C15H11orf96	Chromosome 15 open reading frame, human C11orf96	−2.369	8.58e-03	Downregulated
LRRN3	Leucine rich repeat neuronal 3	−2.312	4.23e-02	Downregulated
CD44	CD44 molecule	−2.176	4.20e-03	Downregulated
ASB5	Ankyrin repeat and SOCS box containing 5	−2.089	2.42e-02	Downregulated
TRIB3	Tribbles pseudokinase 3	−2.085	3.32e-02	Downregulated
SPAG1	Sperm associated antigen 1	−1.948	1.10e-03	Downregulated
HSPB8	Heat shock protein family B (small) member 8	−1.942	4.20e-03	Downregulated
PVR	Poliovirus receptor, transcript variant X2	−1.934	1.94e-03	Downregulated
NUDT4	Nudix hydrolase 4	−1.885	4.23e-02	Downregulated
BMP2K	BMP2 inducible kinase	−1.793	3.19e-02	Downregulated
CNN1	Calponin 1	−1.780	2.36e-05	Downregulated
JUND	JunD proto-oncogene	−1.763	4.23e-02	Downregulated
GEM	GTP binding protein overexpressed in skeletal muscle	−1.615	2.70e-02	Downregulated
RNF115	Ring finger protein 115	−1.553	6.56e-05	Downregulated
EMP1	Epithelial membrane protein 1	−1.541	6.30e-04	Downregulated
TIAM2	T cell lymphoma invasion and metastasis 2, transcript variant X1	−1.378	4.20e-03	Downregulated
SLC1A5	Solute carrier family 1 member 5	−1.375	1.90e-04	Downregulated
SERTAD1	SERTA domain containing 1	−1.231	9.45e-03	Downregulated
GNPTAB	N-acetylglucosamine-1-phosphate transferase alpha and beta subunits	−1.176	6.49e-03	Downregulated
SAMD4A	Sterile alpha motif domain containing 4 A	−1.138	4.87e-02	Downregulated
tRNA-Gln	tRNA	−1.085	7.47e-03	Downregulated
tRNA-Met	tRNA	−1.019	4.04e-02	Downregulated
CCNYL1	Cyclin Y like 1, transcript variant X2	−0.905	2.25e-02	Downregulated
ADCY6	Adenylate cyclase 6	1.283	3.64e-05	Upregulated
LOC112446423	lncRNA	3.396	2.92e-02	Upregulated
LOC617141	Cationic amino acid transporter 3-like, transcript variant X3	5.294	6.30e-04	Upregulated
LOC781412	Pseudogene	6.380	2.40e-11	Upregulated

**Table 4:** List of sirloin muscle DEGs with their statistical and fold change information.

Gene_Symbol	Description	logFC	Adj_P_Value	Expression_Status
LOC101904275	Pseudogene	−4.481	4.03e-02	Downregulated
PPARGC1A	PPARG coactivator 1 alpha	−2.912	1.85e-02	Downregulated
NAB2	NGFI-A binding protein 2	−2.166	2.25e-15	Downregulated
NFATC2	Nuclear factor of activated T cells 2, transcript variant X3	−1.839	3.00e-03	Downregulated
SGMS2	Sphingomyelin synthase 2	−1.670	2.24e-02	Downregulated
MAP2K6	Mitogen-activated protein kinase kinase 6	−1.039	3.00e-03	Downregulated
LOC112448022	lncRNA	−0.839	3.19e-02	Downregulated
STX7	Syntaxin 7	−0.736	2.30e-02	Downregulated
PTPN21	Protein tyrosine phosphatase, non-receptor type 21, transcript variant X2	−0.606	4.03e-02	Downregulated
RIPOR3	RIPOR family member 3, transcript variant X3	1.060	4.00e-04	Upregulated
LOC533307	Tubulin beta-7 chain	1.291	4.00e-04	Upregulated
LOC104970902	lncRNA	2.399	3.26e-02	Upregulated
RGS1	Regulator of G protein signaling 1	2.570	4.76e-02	Upregulated
LOC781412	Incharacterized LOC781412	5.290	3.87e-13	Upregulated
CYP4B1	Cytochrome P450, family 4, subfamily B, polypeptide 1	5.725	1.05e-08	Upregulated

Common DEGs between the two tissues include LOC101904275, PPARGC1A, and SGMS2, which were downregulated in both round and sirloin muscles. Additionally, LOC781412 was found to be upregulated in both tissues. PPARGC1A is involved in adipogenesis and energy homeostasis, while SGMS2 is crucial for lipid metabolism.

In the round muscle, various genes related to myogenesis, adipogenesis, fibrogenesis, and energy homeostasis were significantly downregulated. These include JUND, CNN1, ENAH, TNMD, ASB5, THBS1, PPARGC1A, CD44, GEM, RUNX1, DLK2, and COL11A1. Conversely, genes like ADCY6 were upregulated in the round muscle. Additionally, lncRNAs (LOC104970902 and LOC112446423) and a pseudogene (LOC781412) also showed increased expression. These lncRNAs could be modulating gene expression to optimize muscle growth in response to increased maternal nutrition, potentially affecting meat quality and muscle fiber composition^26^.

In the sirloin muscle, several genes were also significantly downregulated. Notable among them are NFATC2, CYP4B1, and MAP2K6.

### Gene ontology (GO) enrichment analysis

Our study conducted GO analysis on DEGs in the round and sirloin muscles of neonatal Hanwoo calves to explore the effects of maternal overnutrition on muscle development genes. Initially, DEGs were meticulously selected based on an adjusted p-value < 0.05, yielding 43 DEGs for round muscle and 15 DEGs for sirloin muscle. To gain a deeper insight into the biological implications of these DEGs, we expanded our analysis by adopting a less stringent approach, considering DEGs significant at an adjusted p-value < 0.2. This criterion has been applied in some studies to interpret the biological functions of DEGs^12^. Expanding the criteria helps to capture broader biological meanings, especially when initial stringent criteria may overlook significant pathways and processes. This expanded analysis significantly increases the number of DEGs significantely (in total of 90 for round and 30 for sirloin), enabling a more detailed GO analysis across biological processes, molecular functions, and cellular components (Table S2).

**Fig. 3:**
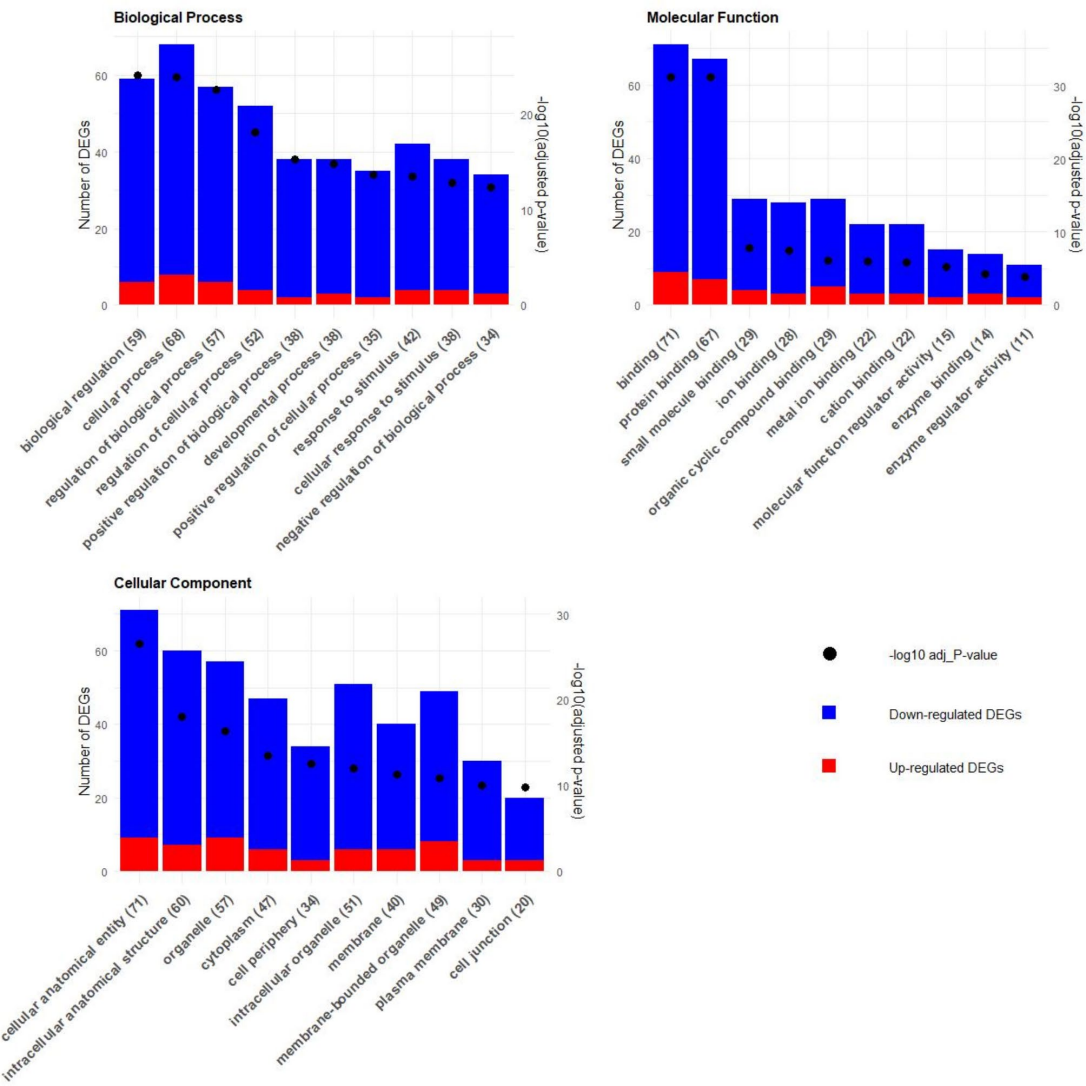
GO enrichment analysis for round muscle tissue: This figure displays the GO enrichment analysis results, highlighting the top 10 terms across biological processes, molecular functions, and cellular components. The Y-axis shows the number of DEGs, with blue and red bars indicating downregulated and upregulated DEGs for each term, respectively. The X-axis lists the top 10 terms. The opposite Y-axis depicts the -log10 of the adjusted p-value for each term, represented by dots.

In both round and sirloin muscle tissues, maternal overnutrition significantly impacted biological processes, molecular functions, and cellular components linked to myogenesis, adipogenesis, and energy regulation. In the round muscle, the most relevant biological process GO terms were “biological regulation” (GO:0065007), “cellular process” (GO:0009987), “regulation of biological process” (GO:0050789), “regulation of cellular process” (GO:0050794), and “response to stimulus” (GO:0050896). These terms were associated with key genes such as PPARGC1A, THBS1, FOSL1, RUNX1, CD44, and SGMS2, which were notably downregulated. Similarly, the significant molecular function GO terms included “binding” (GO:0005488), “protein binding” (GO:0005515), “small molecule binding” (GO:0036094), “ion binding” (GO:0043167), and “organic cyclic compound binding” (GO:0097159). These molecular function terms were linked to key downregulated genes like PPARGC1A, THBS1, FOSL1, RUNX1, CD44, and SGMS2, while upregulated genes such as ADCY6, CCNB2, and TELO2 were also highlighted. For cellular components, significant GO terms included “cellular anatomical entity” (GO:0110165), “intracellular anatomical structure” (GO:0005622), “organelle” (GO:0043226), “cytoplasm” (GO:0005737), and “cell periphery” (GO:0071944). Key upregulated genes were ADCY6, CYP4B1, ME2, while downregulated genes included THBS1, FOSL1, SGMS2, RUNX1, and CD44 (Fig. 3; Table S2).

**Fig. 4:**
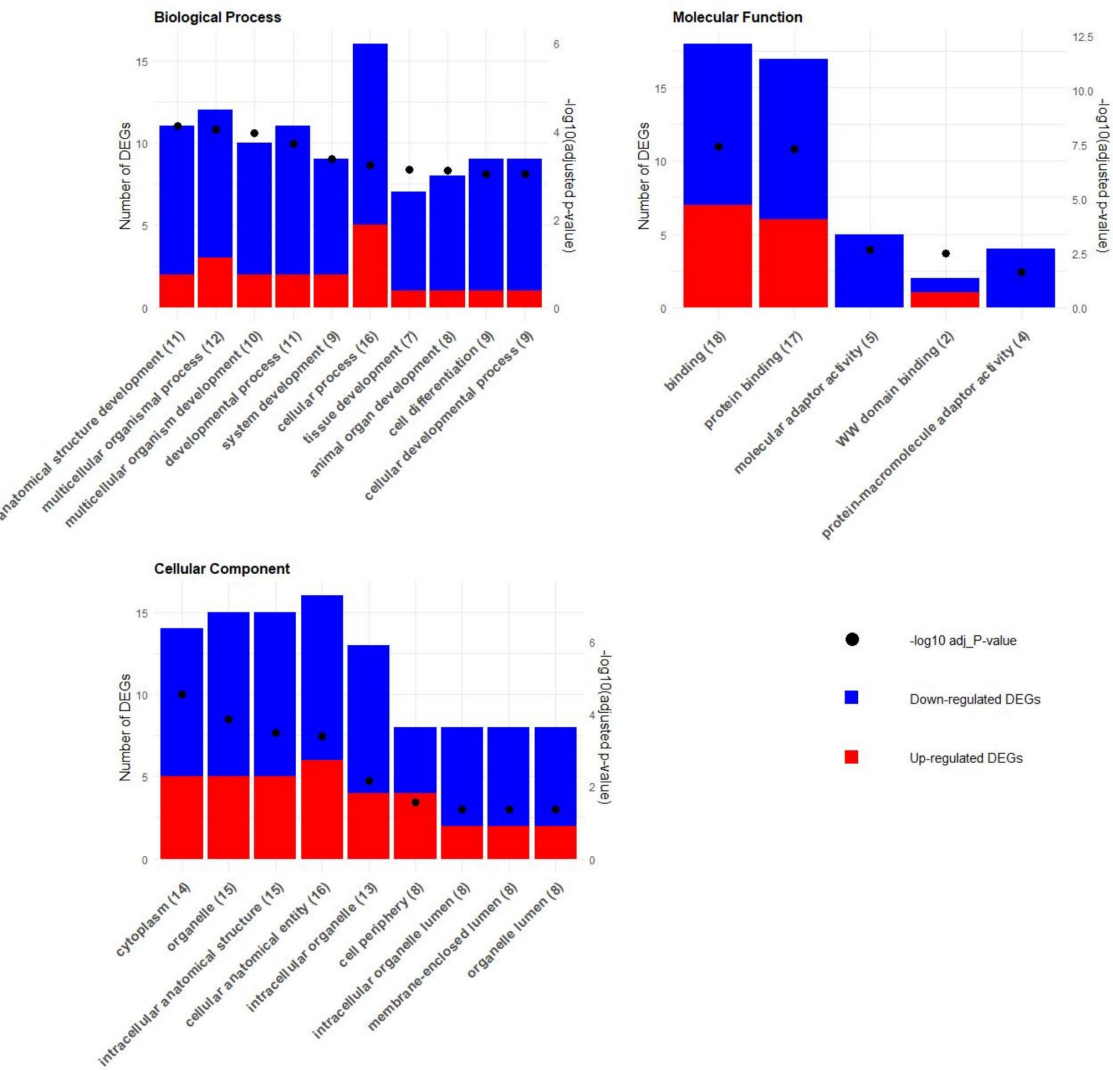
GO enrichment analysis for sirloin muscle tissue: This figure displays the GO enrichment analysis results, highlighting the top 10 terms across biological processes, molecular functions, and cellular components. The Y-axis shows the number of DEGs, with blue and red bars indicating downregulated and upregulated DEGs for each term, respectively. The X-axis lists the top 10 terms. The opposite Y-axis depicts the -log10 of the adjusted p-value for each term, represented by dots.

In the sirloin muscle, maternal overnutrition also significantly impacted similar biological processes. The most significant biological process GO terms included “anatomical structure development” (GO:0048856), “multicellular organismal process” (GO:0032501), “developmental process” (GO:0032502), “cellular process” (GO:0009987), and “cell differentiation” (GO:0030154). These terms were associated to crucial genes such as PPARGC1A, NAB2, ENAH, NFATC2, SGMS2, and KLF5, which were significantly downregulated, and MMP2, which was upregulated. For molecular function, the significant GO terms were “binding” (GO:0005488), “protein binding” (GO:0005515), and “molecular adaptor activity” (GO:0060090). These terms were associated with key downregulated genes such as PPARGC1A, NAB2, and ENAH. Upregulated genes like MMP2 and RIPOR3 were also significantly enriched in these terms. In terms of cellular component, the top GO terms affected were “cytoplasm” (GO:0005737), “organelle” (GO:0043226), and “intracellular anatomical structure” (GO:0005622). These terms involved crucial genes such as PPARGC1A, ENAH, NFATC2, SGMS2, and KCNB1. Upregulated genes like MMP2 and SHISA5 also played significant roles (Fig. 4; Table S2).

Across both muscle tissues, maternal overnutrition had a profound effect on key genes and biological processes, particularly those associated with myogenesis, adipogenesis, and energy regulation. Genes such as PPARGC1A and SGMS2 were consistently impacted, highlighting their importance energy metabolism and muscle development.

### KEGG pathway enrichment analysis

**Fig. 5:**
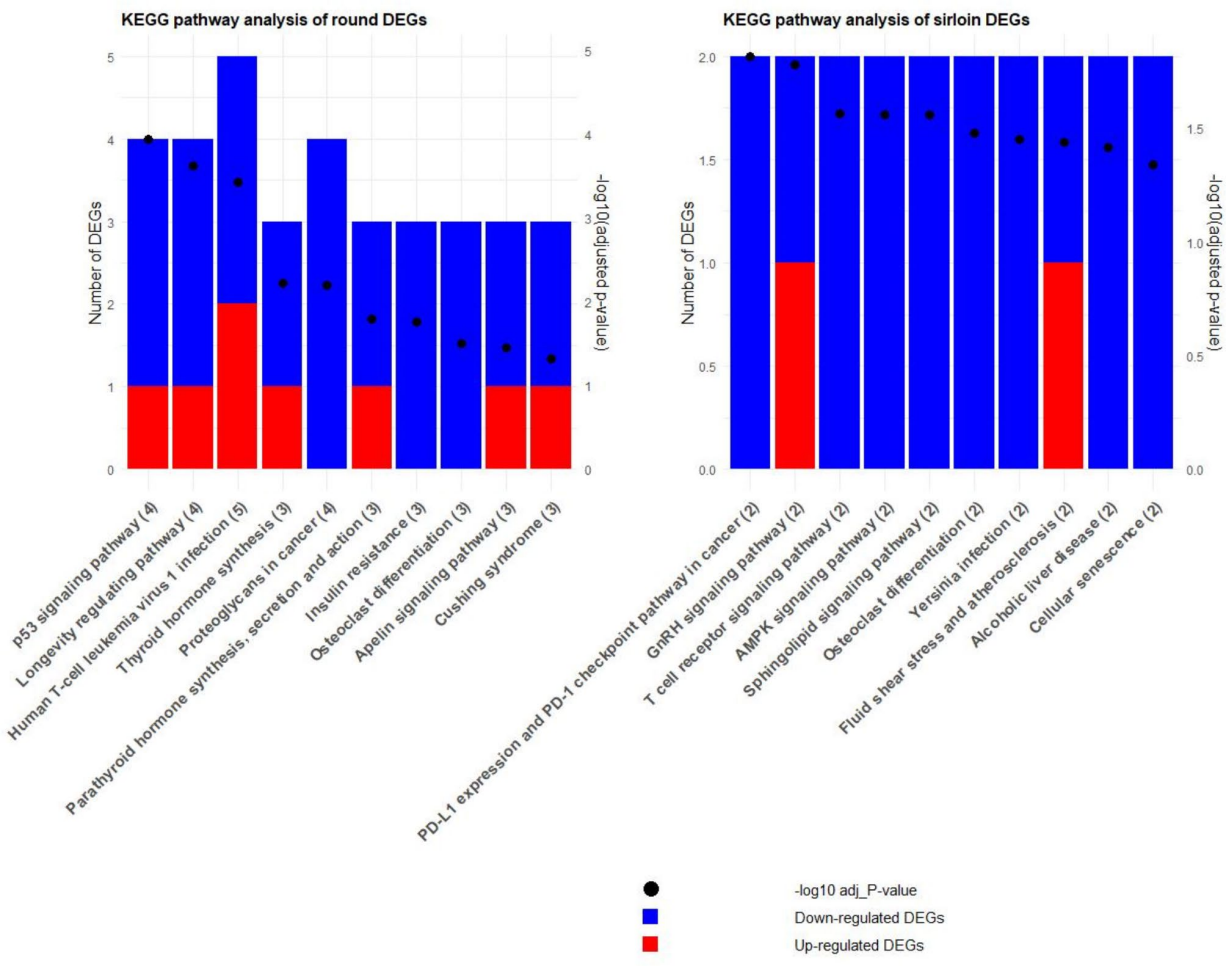
KEGG pathway analysis for round and sirloin muscle tissues: This figure displays the KEGG pathways analysis results, highlighting the top 10 terms. The Y-axis shows the number of DEGs, with blue and red bars indicating downregulated and upregulated DEGs for each term, respectively. The X-axis lists the top 10 terms. The opposite Y-axis depicts the -log10 of the adjusted p-value for each term, represented by dots.

KEGG pathways analysis was conducted to explore the roles of genes with altered expression in the round and sirloin muscles of neonatal Hanwoo calves. Similar to the GO analysis, adopting a broader criterion (adjusted p-value < 0.2) allowed us to identify some pathways, in the round and the sirloin muscles (Table S3). Figure 5 illustrates the top 10 KEGG pathways for round and sirloin muscles of Hanwoo neonatal calves.

In the round muscle tissue of neonatal Hanwoo calves, maternal overnutrition significantly impacted pathways associated with myogenesis, adipogenesis, and energy regulation. The most relevant KEGG pathways include “p53 signaling pathway” (KEGG:04115), “Longevity regulating pathway” (KEGG:04211), “Human T-cell leukemia virus 1 infection” (KEGG:05166), “Thyroid hormone synthesis” (KEGG:04918), and “Proteoglycans in cancer” (KEGG:05205). These pathways involved key upregulated genes such as ADCY6 and CCNB2, and downregulated genes like PPARGC1A, CREB5, THBS1, and CD44, which play crucial roles in muscle development and metabolic processes. Additionally, pathways such as “Insulin resistance” (KEGG:04931) and “Apelin signaling pathway” (KEGG:04371) were significantly enriched, highlighting the complex regulatory response to maternal overnutrition in muscle tissues (Fig. 5; Table S3).

In the sirloin muscle tissue, maternal overnutrition also notably influenced pathways related to myogenesis, adipogenesis, and energy regulation. The most significant KEGG pathways were “PD-L1 expression and PD-1 checkpoint pathway in cancer” (KEGG:05235), “GnRH signaling pathway” (KEGG:04912), “T cell receptor signaling pathway” (KEGG:04660), “Sphingolipid signaling pathway” (KEGG:04071), and “AMPK signaling pathway” (KEGG:04152). These pathways were associated with key downregulated genes such as NFATC2, PPARGC1A, and MAP2K6. Upregulated genes like MMP2 were also significantly enriched in these terms, indicating their role in muscle development and metabolic processes (Fig. 5; Table S3). It is important to note that the number of genes included in these pathways was relatively small, which may limit the comprehensiveness of the pathway analysis.

### Protein-protein interaction (PPI) analysis of round and sirloin muscle tissues

**Fig. 6:**
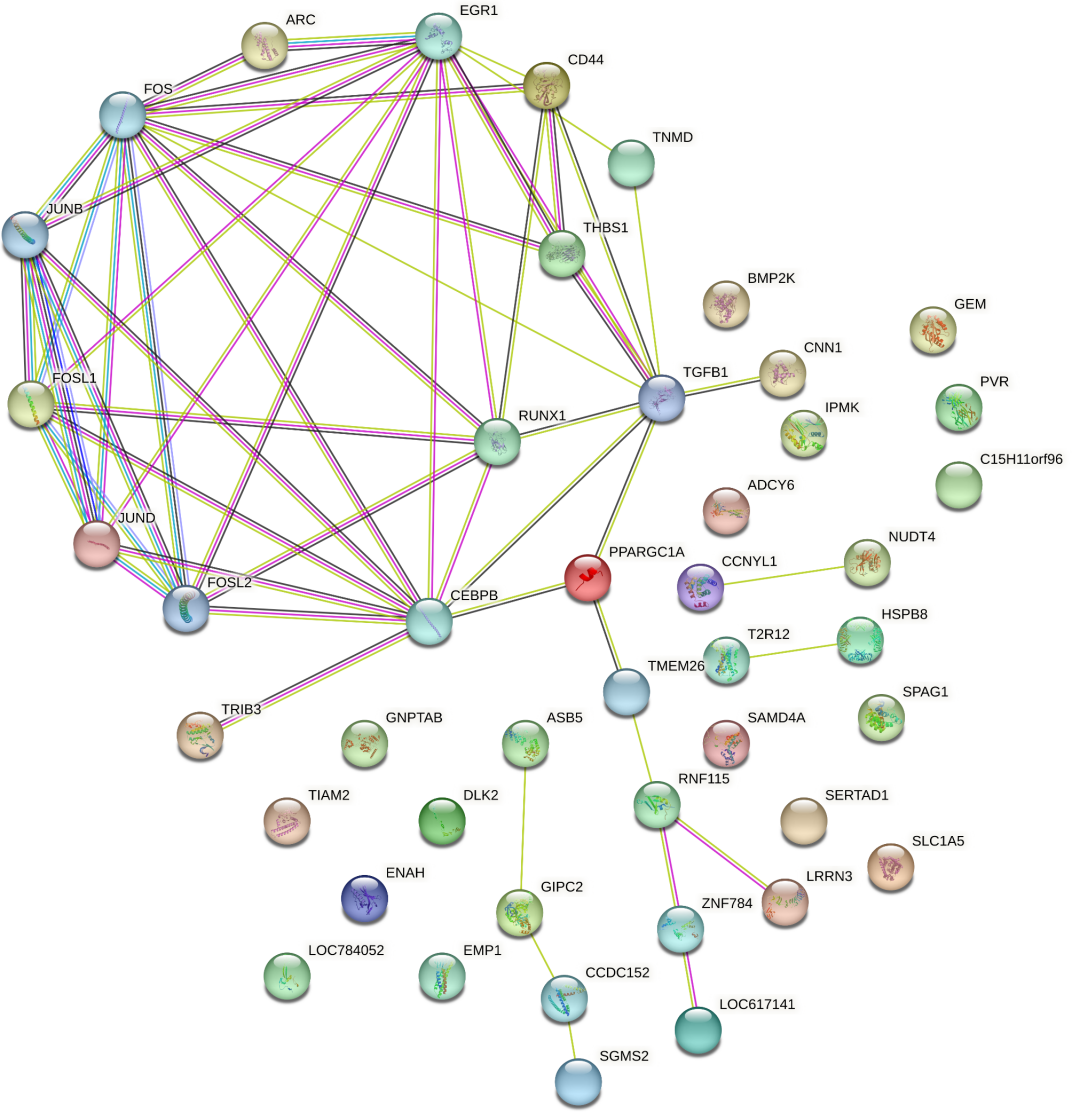
Protein-Protein Interaction (PPI) Network for Round Muscle DEGs. This figure depicts the PPI network of differentially expressed genes (DEGs) in the round muscle tissue of neonatal Hanwoo calves subjected to maternal overnutrition. The network consists of 45 nodes and 56 edges, highlighting significant interactions (PPI enrichment p-value: 5.24e-13).

In the round muscle tissue of neonatal Hanwoo calves subjected to maternal overnutrition, the PPI network revealed a significantly enriched interaction landscape (PPI enrichment p-value: 5.24e-13), indicating strong interconnectedness among the DEGs. Key downregulated genes such as PPARGC1A, THBS1, FOSL1, RUNX1, and CD44, played central roles in the network (Fig. 6), interacting with multiple other genes involved in myogenesis and adipogenesis. Notably, the upregulated gene ADCY6 also formed crucial interactions, suggesting a compensatory mechanism in response to overnutrition. The network statistics highlighted 45 nodes and 56 edges, with an average node degree of 2.49 and a local clustering coefficient of 0.46, demonstrating the complexity and significance of these interactions in muscle tissue development and energy regulation.

**Fig. 7:**
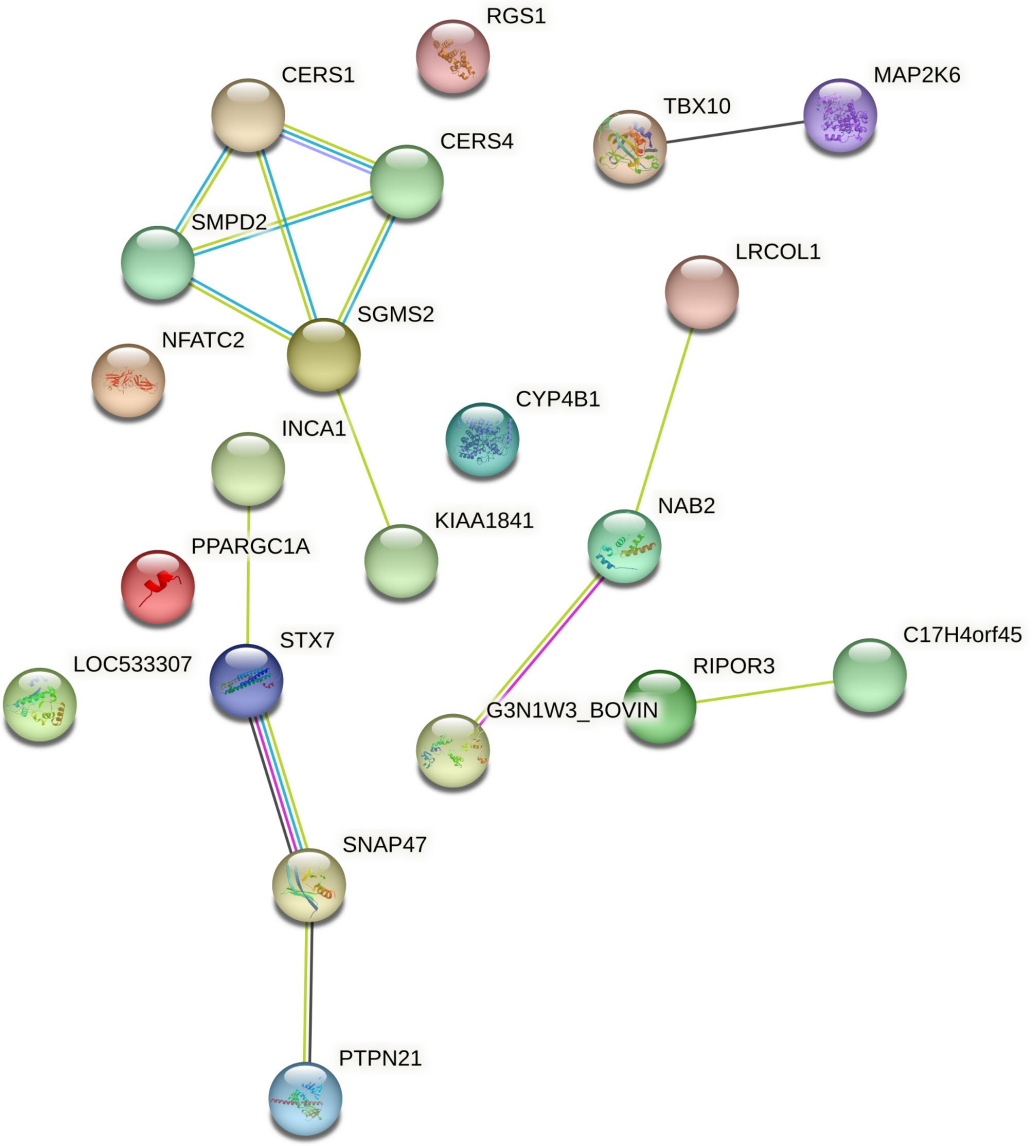
Protein-Protein Interaction (PPI) Network for Sirloin Muscle DEGs. This figure illustrates the PPI network of DEGs in the sirloin muscle tissue of neonatal Hanwoo calves exposed to maternal overnutrition. The network includes 21 nodes and 14 edges, with fewer significant interactions (PPI enrichment p-value: 0.176).

In contrast, the sirloin muscle tissue displayed a less interconnected PPI network (PPI enrichment p-value: 0.176), suggesting fewer significant interactions among the DEGs. Key downregulated genes such as PPARGC1A, NFATC2, and MAP2K6 were central in this network, while the upregulated gene like RIPOR3 showed notable interactions (Fig. 7), emphasizing their roles in muscle and metabolic processes. This network consisted of 21 nodes and 14 edges, with an average node degree of 1.33 and a local clustering coefficient of 0.595. These results reflect the differential impact of maternal overnutrition on muscle tissues, highlighting a more pronounced interaction network in round muscle compared to sirloin muscle.

### Random forest analysis

**Fig. 8:**
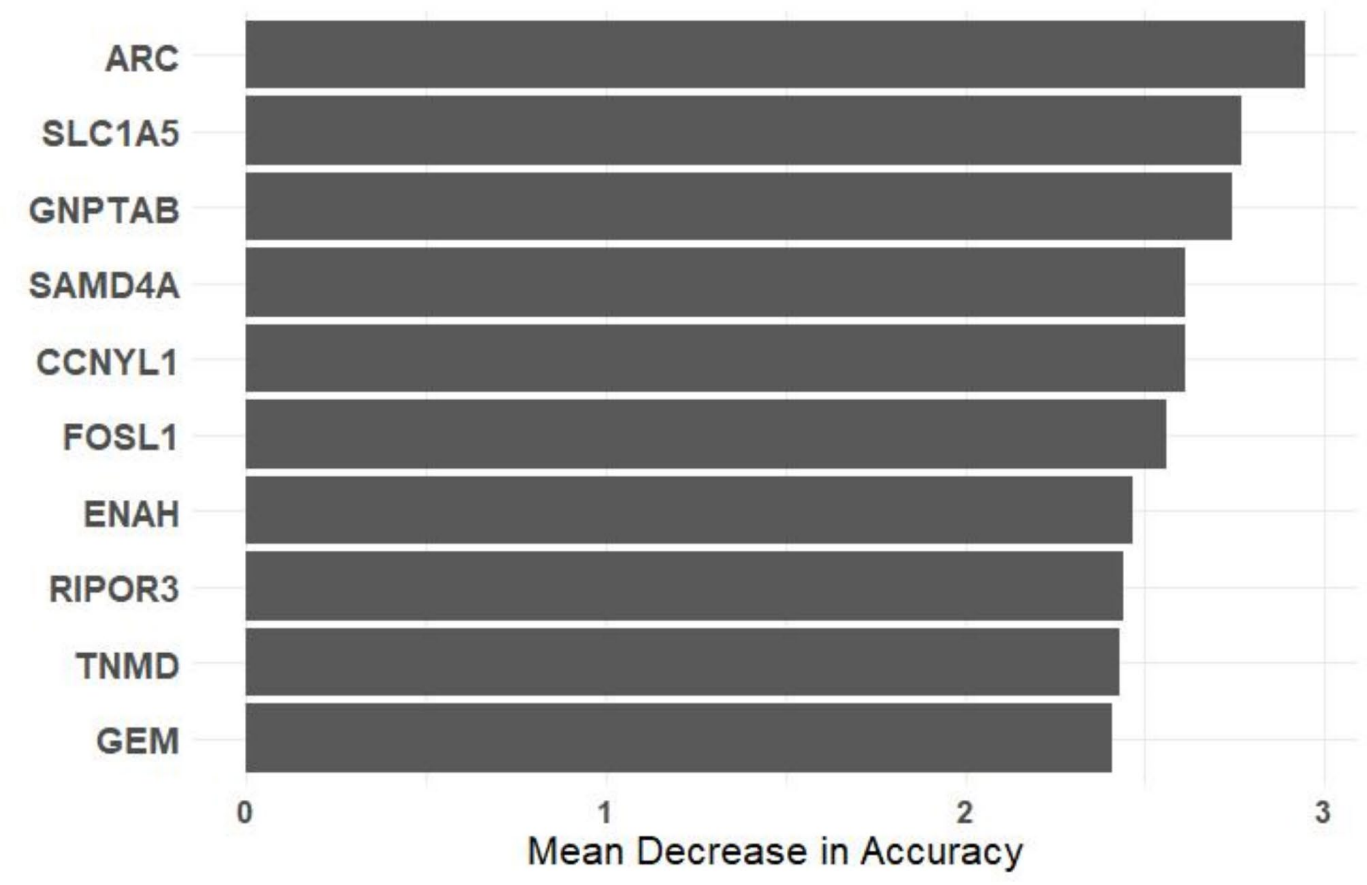
Top 10 genes from random forest classification. This figure displays the genes identified by Random Forest classification as the most important based on their impact on model accuracy. The y-axis lists the gene names, and the x-axis shows the mean decrease in accuracy (MDA) associated with each gene.

The random forest analysis on the 54 DEGs from the round and sirloin muscle tissues of neonatal Hanwoo calves identified the top 10 genes crucial for distinguishing between different groups based on their expression patterns. The analysis highlighted ARC, SLC1A5, GNPTAB, SAMD4A, CCNYL1, FOSL1, ENAH, RIPOR3, TNMD, and GEM as the most important genes, ranked by their Mean Decrease in Accuracy (Fig. 8). This metric indicates the significance of each gene in maintaining the model’s accuracy, with higher values reflecting greater importance. ARC, SLC1A5, GNPTAB, SAMD4A, CCNYL1, FOSL1, and ENAH, showed the highest importance scores. This methodological approach helps identify genes that may play a role in differentiating the effects of overnutrition, offering preliminary insights into potential molecular pathways involved in fetal muscle development in response to maternal dietary conditions.

### The validation of RNA-seq data by qPCR (quantitative polymerase chain reaction)

**Fig. 9:**
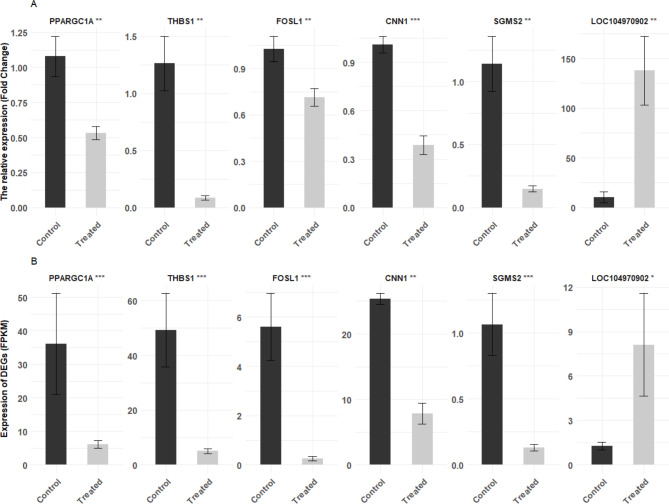
Validation of RNA-Seq data using qPCR. (**A**) Shows the validation of six DEGs by qPCR, with fold changes in expression shown separately for round muscle (PPARGC1A, THBS1, FOSL1, CNN1; left panel) and sirloin muscle (SGMS2, LOC104970902; right panel). (**B**) Depicts FPKM values from RNA-Seq for the same six DEGs in round and sirloin muscles of neonatal calves. The y-axis indicates fold change for qPCR and gene expression level (FPKM) for RNA-Seq, while the x-axis lists the groups compared. Above the bars, gene names and their respective p-value symbols from qPCR results are displayed in (A): *** (*p* < 0.001), ** (*p* < 0.01), * (*p* < 0.05). In (**B**), gene names and their respective adjusted p-value symbols from RNA-Seq results are displayed: *** (adj *p* < 0.001), ** (adj *p* < 0.01), * (adj *p* < 0.05).

To validate the alignment of mRNA expression levels with RNA sequencing data, we employed qPCR to quantify mRNA concentrations of selected DEGs. Six DEGs were chosen based on their significant differential expression, with four from the round muscle and two from the sirloin muscle. The qPCR results indicated that in the round muscle, PPARGC1A, THBS1, FOSL1, and CNN1 expression levels were significantly lower in the treated group compared to the control group (Fig. 9A, left panel). Conversely, in the sirloin muscle, LOC104970902 expression levels were significantly higher, while SGMS2 expression levels were significantly lower in treated calves compared to controls (Fig. 9A, right panel). The RNA-seq analysis showed consistent mRNA expression levels (Fig. 9B). This consistency between the two methods confirms the reliability and robustness of our sequencing data.

## Discussions

Muscle development, an important economic trait, is influenced by genetic and environmental factors. Maternal nutrition during pregnancy significantly impacts the offspring growth, productivity, and health^9,27,28^. This study examines the impact of maternal overnutrition during mid-to-late pregnancy on gene expression in the round and sirloin muscles of neonatal Hanwoo calves. Through RNA-seq and qPCR, we identified significant changes in genes related to myogenesis, adipogenesis, and energy metabolism, expanding upon previous research on maternal nutrition and fetal development.

Our results revealed both common and unique patterns of gene expression associated with muscle development and energy homeostasis in the round and sirloin muscles of neonatal calves. Gene expression in both muscle types predominantly showed downregulation. Common DEGs, such as LOC101904275, PPARGC1A, and SGMS2, were downregulated in both the round and sirloin muscles, suggesting these genes have universal roles in muscle development and energy regulation across muscle types. Additionally, LOC781412 was found to be upregulated in both tissues, indicating a shared response to maternal overnutrition. However, we also observed muscle-specific differences. Genes like RUNX1 and CD44 were downregulated in the round muscle but not in the sirloin, highlighting the distinct functional roles and metabolic demands of each muscle. The round muscles, such as the *Semimembranosus* and *Biceps femoris*, are primarily composed of oxidative type I fibers that are adapted for sustained activities, indicating a different nutritional requirement and adaptive response compared to the sirloin muscles like the *Gluteus medius* and *Psoas major*, which contain more glycolytic type II fibers suited for rapid, powerful movements. These fiber type differences likely contribute to the distinct gene expression patterns observed in response to maternal overnutrition, impacting myogenesis and muscle development differently in each muscle type^29^. The specific nutritional needs of each muscle type, particularly in response to maternal energy and protein intake, could be crucial for optimizing calf growth and potentially enhancing body weight through tailored nutritional strategies^12,30^. Understanding these muscle-specific responses is essential for developing interventions that support healthy muscle development and metabolic function in offspring, especially in livestock where muscle composition directly affects meat quality and economic value. The heterogeneity of skeletal muscles and their variable response to stimuli, including susceptibility to atrophy or hypertrophy^30^, further underline the importance of understanding muscle biology in response to environmental and genetic factors.

While maternal undernutrition has been extensively studied, the effects of overnutrition remain less understood. However, both nutritional extremes can influence fetal programming, potentially altering muscle development and meat quality^31^. Previous studies have indicated that maternal nutrition can significantly influence the developmental programming of offspring, affecting muscle development^30^and metabolic health^16,32^. Our study adds to this knowledge by specifically examining the impact of maternal overnutrition on the transcriptome profile of round and sirloin muscles in Hanwoo neonatal calves. Although direct research on maternal overnutrition’s effects on neonatal calf muscle development is limited, our findings align with broader studies on maternal nutrition’s impact on muscle development. For example, pre- and post-parturition nutritional adjustments, including protein and energy supplementation, have been shown to downregulate genes linked to muscle development and differentiation in sirloin muscle of calves^12^.

Studies have found that maternal protein or fat supplementation during mid-gestation in cattle and sheep can enhance offspring muscle development and metabolic responses, highlighting the nuanced roles of specific nutrients during critical developmental windows^33,34^. Maternal supplementation during mid-gestation increased mRNA expression of genes like FGF2 and PPARα in skeletal muscle but did not affect myofiber numbers^18^. High maternal nutrition during mid-gestation increased Preadipocyte Factor-1 (PREF-1) but did not significantly change other adipogenesis and myogenesis genes^11^. In addition, maternal protein led to the differential expression of 310 genes, with a notable upregulation in genes linked to insulin signaling and apoptosis^15^.

Maternal nutrition significantly influences fetal skeletal muscle development by affecting mesenchymal stem cell differentiation into myocytes, adipocytes, and fibroblasts, which are essential for muscle mass, marbling, and collagen deposition^16,30,31^. This study explores whether the DEGs observed in the round and sirloin muscles of neonatal calves, as a result of maternal overnutrition, are associated with myogenesis, adipogenesis, fibrogenesis and energy metabolism.

Our analysis revealed significant downregulation of key myogenesis-related genes, such as JUND, CNN1, ENAH, and RUNX1 in the round muscle, and NFATC2 in the sirloin muscle. The downregulation of these genes suggests that maternal overnutrition may impair myogenesis by disrupting the molecular pathways necessary for muscle fiber development and differentiation. GO analysis highlighted terms like ‘biological regulation’, ‘cellular process’, ‘regulation of biological process’, ‘regulation of cellular process’, and ‘response to stimulus’, underscoring these genes’ roles in muscle development. For instance, JUND’s contribution to bovine muscle tissue development^35^and CNN1 stabilizes the actin cytoskeleton and regulates muscle contractility^36^. It is significantly upregulated in intramuscular fat, indicating its role in marbling^37^, and is linked to meat quality traits in Nelore cattle^38^and feed efficiency^39^. Our observed downregulation aligns with decreased CNN1 expression due to maternal feed restriction^40^. ENAH has a role in controlling cell motility and adhesion through cytoskeletal regulation^41^. RUNX1 influences muscle tissue development and repair processes^42,43^. NFATC2 is activated in newly formed myotubes and plays a role in their growth by regulating the addition of myonuclei and increasing the size of myotubes^44,45^. Moreover, KEGG pathway analysis identified pathways related to muscle development and metabolic processes, such as the “p53 signaling pathway” and “Longevity regulating pathway” in the round muscle, and the “GnRH signaling pathway” and “T cell receptor signaling pathway” in the sirloin muscle. These pathways involve key genes like PPARGC1A, THBS1, FOSL1, RUNX1, CD44, and SGMS2, suggesting that altered expression of these genes may impair muscle growth and increase susceptibility to metabolic dysregulation. PPI network analysis highlighted the interconnected roles of these genes. In round muscle, key downregulated genes like PPARGC1A, THBS1, FOSL1, RUNX1, and CD44 played central roles by interacting with genes involved in myogenesis. potentially contributing to reduced muscle development. Notably, the upregulated gene ADCY6 also formed crucial interactions, suggesting a compensatory mechanism in response to overnutrition. ADCY6 encodes adenylate cyclase 6, which converts ATP to cyclic AMP (cAMP), activating protein kinase A (PKA) and enhancing glucose production and lipid metabolism to meet increased energy demands^46^. The upregulation of ADCY6 might indicate an adaptive response to increased energy requirements, possibly as a compensatory mechanism for impaired myogenesis.

Likewise, an upregulation of lncRNAs such as LOC104970902 (sirloin), LOC112446423 (round), and the pseudogene LOC781412 (round and sirloin) were observed. These lncRNAs may modulate gene expression to optimize muscle growth in response to increased maternal nutrition, potentially affecting meat quality and muscle fiber composition^26^. Furthermore, GO analysis showed mostly downregulation in the protein binding term of molecular function. This suggests potential changes in muscle integrity and function, reflecting reduced protein interaction dynamics. Such changes could indicate altered muscle protein turnover, which is critical for maintaining myo-fiber size and function. Protein turnover is critical for myo-fiber size and function, as the balance between protein biosynthesis and degradation is crucial for muscle cell size and functionality^47^. Collectively, these findings suggest that maternal overnutrition has differential effects on genes related to muscle development or myogenesis in the round and sirloin muscles of neonatal calves. As previously reported, maternal overnutrition does not significantly affect muscle fiber numbers^18^. These results align with studies indicating that late pregnancy nutritional supplementation may not significantly enhance myogenesis in fetal skeletal muscle^3^.

In the context of adipogenesis, our analysis revealed a downregulation in several key genes such as TNMD, ASB5, THBS1, PPARGC1A, CD44, and GEM in the round muscle, and CYP4B1 and MAP2K6 in the sirloin muscle. TNMD is downregulated in bovine muscle under higher mineral concentrations, indicating its sensitivity to nutrient levels and mineral homeostasis^48^. It is also induced early in adipocyte differentiation, playing a crucial role in adipogenesis and muscle development, regulated by adipogenic transcription factors such as C/EBPα and PPARγ^49^. ASB5 has been identified as a gene that significantly influences adipogenesis and, by extension, beef quality^50^. Similarly, THBS1 plays a critical role in regulating intramuscular fat deposition post-castration^51^, which not only affects the water holding capacity of meat^52^but also its overall quality in beef cattle. PPARGC1A, downregulated in both round and sirloin muscles, is involved in lipid metabolism, interacting with pathways critical for lipolysis in adipocytes, adipocytokine signaling, and the PPAR signaling pathway^42,53^. The CD44 gene, known for its importance in bovine mammary epithelial cells, also emerges as a crucial regulator of lipid metabolism^54^. CD44 is present on the surface of human adipose stem cells, indicating a potential role in the pluripotency and differentiation of preadipocytes^55^. GEM promotes adipogenesis in goat intramuscular preadipocytes by facilitating lipid accumulation and enhancing the expression of key adipogenic markers such as C/EBPα, C/EBPβ, LPL, PPARγ, and SREBP1^56^. CYP4B1 is significantly more expressed in intramuscular fat compared to subcutaneous fat in cattle, playing a crucial role in cholesterol metabolism and influencing fat deposition and muscle development^57^.​ MAP2K6, linked to intramuscular fat in Nelore cattle^58^, regulates lipid metabolism in Hanwoo cattle^43^. These results contrast with other findings showing that maternal overnutrition increases or has no effect on the levels of adipogenic marker mRNA in the skeletal muscle of fetal beef cattle^3,16^.

Regarding fibrogenesis, we found a downregulation of DLK2 and COL11A1 in round muscle. GO analysis highlighted terms related to anatomical structure development, multicellular organismal process, and cell differentiation, emphasizing their roles in fibrogenesis. DLK2 modulates progenitor cell proliferation and differentiation through interactions with extracellular matrix components and signaling pathways^59^. COL11A1, part of collagen XI, influences meat tenderness by affecting connective tissue integrity in beef cattle^60^. The downregulation of these fibrogenesis-related genes suggests potential alterations in connective tissue development, which could impact muscle quality and function in neonatal calves exposed to maternal overnutrition.

Overall, our findings suggest that overnutrition during the mid to late stages of pregnancy may not significantly enhance myogenesis and adipogenesis in the muscle tissues of neonatal Hanwoo calves. This conclusion is based on the significant downregulation of genes crucial for these developmental processes. For instance, in the round muscle, we observed downregulation of genes such as PPARGC1A, SGMS2, RUNX1, and CD44. Similarly, in the sirloin muscle, genes like NFATC2 and MAP2K6, which are important for muscle development and response to metabolic changes, were also downregulated. These patterns suggest that maternal overnutrition leads to a suppression of genes involved in myogenesis and adipogenesis rather than an enhancement, highlighting a potential inhibitory effect on muscle and fat cell development pathways in neonatal calves.

Maternal nutrition during pregnancy significantly influences offspring’s skeletal muscle energy metabolism, leading to adaptations such as reduced glucose transport and ATP production, and diminished transcriptional metabolic flexibility. These adaptations can predispose offspring’s muscle to favor fatty acids over carbohydrates for ATP synthesis, even with ample glucose availability^61^, affecting marbling in beef animals fed high-energy diets. In this study, PPARGC1A, a crucial gene in this domain, was downregulated in both muscle tissues. This downregulation suggests an inhibition of its role as a transcriptional coactivator that regulates genes involved in these metabolic processes, especially in metabolically active tissues^42,43^. Conversely, ADCY6 were upregulated in round of Neonatal calves, respectively. ADCY6 has a function in glucose production^62^. Similarly, the upregulation of RGS1 in the sirloin muscle indicates its involvement in cellular energy regulation mechanisms. RGS1 is responsible for decreasing the activity of G-protein signaling by binding to the α subunit of G proteins to increase the conversion of guanosine triphosphate (GTP) to guanosine diphosphate (GDP)^63^, suggesting its involvement in cellular energy regulation mechanisms. GO analysis highlighted significant terms related to “biological regulation” and “response to stimulus,” which encompass metabolic processes and energy regulation. These findings underscore the complex interplay between maternal nutrition, gene expression, and metabolic outcomes, highlighting the need for further research to understand the long-term effects of maternal overnutrition on offspring health and development.

The Random Forest analysis in our study highlighted the significance of several genes in the context of muscle development and energy homeostasis in neonatal Hanwoo calves, emphasizing their potential impact on meat quality. Among the genes identified, ARC, was noted as the most influential gene. ARC plays a crucial role in muscle development by regulating cytoskeletal dynamics and processes such as actin polymerization, essential for muscle fiber formation and maintenance^64^. This regulation is vital for proper muscle growth, suggesting that alterations in ARC expression could impact muscle structure and function, potentially affecting meat quality and yield. SLC1A5, the second key gene, is a significant transcription target of the TEAD1 gene, with its expression crucial for the proliferation and differentiation of myoblasts during embryonic skeletal muscle development^65^. The involvement of SLC1A5 in myoblast differentiation underscores its importance in early muscle development stages. However, previous research has indicated that while TEAD1 regulates muscle growth via SLC1A5, maternal dietary restriction during pregnancy does not significantly affect SLC1A5 expression^66^​. This finding suggests that SLC1A5 may be differentially regulated under conditions of overnutrition compared to undernutrition, pointing to a complex regulatory mechanism that could affect muscle development and metabolism differently depending on the nutritional environment. GNPTAB as the third gene, involved in the formation of mannose 6-phosphate recognition markers on lysosomal enzymes^67^, is linked to metabolic pathways and cellular processes crucial for muscle development. Disruptions in GNPTAB expression may influence muscle tissue homeostasis and metabolic efficiency, potentially leading to altered muscle growth and composition in offspring exposed to maternal overnutrition.

To sum up, this study demonstrates that maternal overnutrition during mid-to-late pregnancy can lead to the downregulation of key genes involved in myogenesis, adipogenesis, fibrogenesis, and energy metabolism in neonatal Hanwoo calves. These findings suggest that maternal overnutrition not only impairs muscle development but may also predispose offspring to metabolic dysregulation, potentially affecting muscle quality and the overall health of the calves.

## Conclusions

This study demonstrates that maternal overnutrition during mid-to-late pregnancy significantly impacts gene expression in the round and sirloin muscles of neonatal Hanwoo calves, primarily leading to the downregulation of genes crucial for muscle development, adipogenesis, fibrogenesis, and energy metabolism. Key genes, such as PPARGC1A, THBS1, ENAH, RUNX1, CD44, JUND, NFATC2, and MAP2K6, were downregulated, suggesting a suppression of pathways involved in muscle cell differentiation and metabolic regulation. These molecular changes may indicate a potential reduction in muscle fiber formation and a limitation in fat deposition rather than promoting increased adipose tissue, challenging the typical expectations of overnutrition effects. The identified changes in gene expression profiles and associated pathways highlight the complex regulatory mechanisms in muscle development and metabolism influenced by maternal nutrition. Additionally, our Random Forest analysis revealed genes like ARC, SLC1A5, and GNPTAB as influential in these processes, suggesting new targets for future research aimed at optimizing prenatal nutrition strategies. These findings underscore the importance of balanced maternal diets to support optimal muscle development and metabolic health in offspring, with potential implications for improving growth performance and meat quality in beef cattle.

## Electronic supplementary material

Below is the link to the electronic supplementary material.

Supplementary Material 1

Supplementary Material 2

Supplementary Material 3

Supplementary Material 4

Supplementary Material 5

Supplementary Material 6

## Data Availability

All original findings of the study are included within the article and its Supplementary Material. The RNA-seq read data generated and analyzed during this study are available in the Gene Expression Omnibus (GEO) repository, under the accession number GSE275560, as part of BioProject PRJNA1151676.
